# Technology, connection, and engagement—achieving a balance for maximal value hybrid conferences in radiology

**DOI:** 10.1007/s00247-025-06187-5

**Published:** 2025-02-17

**Authors:** Amanda Liu, Julian Lopez-Rippe, Janet Reid

**Affiliations:** 1 Department of Radiology & Biomedical Imaging, University of California, San Francisco, 1975 4th St., San Francisco, CA 94158 USA; 2 Department of Radiology, Children’s Hospital of Philadelphia, Perelman School of Medicine, University of Pennsylvania, 3401 Civic Center Blvd, Philadelphia, PA 19104 USA; 3 University of Pennsylvania, Philadelphia, USA

**Keywords:** Problem-based learning, Motivation, Pandemics, COVID-19, Radiology, Medical education, Internet, Technology

## Abstract

**Introduction:**

The COVID-19 pandemic catalyzed a transformation in medical education, leading to the emergence of hybrid learning formats that combine in-person and remote participation. While this format offers increased flexibility and accessibility, it presents unique challenges for both educators and learners in radiology training programs.

**Methods:**

This review examines the critical elements necessary for successful hybrid resident and fellow conferences in radiology education, focusing on three key domains: technology, connection, and engagement.

**Results:**

Technical considerations, including appropriate audiovisual setup and the designation of conference moderators, are crucial for seamless integration of remote participants. Digital tools such as audience response systems, web-based DICOM viewers, and collaborative platforms can enhance interactivity and simulate clinical practice effectively in the hybrid format. Creating an effective learning environment requires careful attention to establishing expectations, maintaining psychological safety, and ensuring equal participation between in-person and remote attendees. Active learning strategies, such as case-based discussions, buzz groups, and peer instruction, can be adapted to include both remote and in-person learners effectively.

**Conclusion:**

While the flexibility of hybrid education offers advantages for work-life balance and accessibility, maintaining educational quality requires thoughtful implementation of these strategies. As hybrid conferences become a permanent fixture in radiology education, programs must address these challenges to optimize the learning experience for all participants while preserving the essential elements of traditional radiology training.

**Graphical Abstract:**

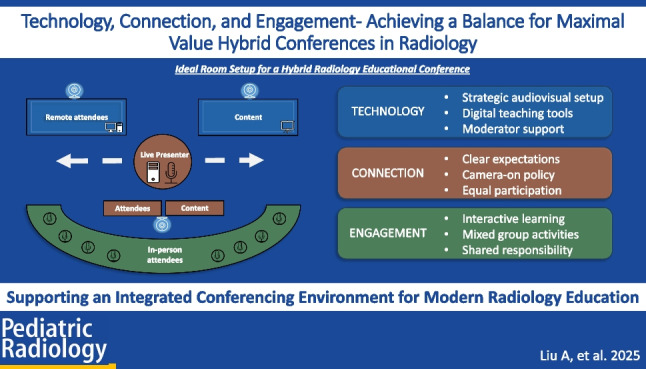

## Introduction

The COVID−19 pandemic transformed education, for better or for worse. The initial response to social distancing requirements was to transition to online-only remote education wherever possible, using video teleconferencing platforms. As public health restrictions eased, in-person educational activities gradually resumed. However, realization of the benefits of online education has resulted in a third “hybrid” educational format, in which some learners, as well as educators, attend in person and some attend remotely.

While the initial shift to online education after COVID-19 was out of necessity, subsequent studies identified certain benefits to online education. Accessibility and flexibility are cited by both learners and faculty as the primary advantages of online education [1–5]. Residents and fellows from multiple specialties surveyed on the virtual conference format have reported increased time for sleep and wellness and improved work-life balance with a transition to online didactics during the COVID-19 pandemic. Specifically, they appreciate saving time on commuting or being able to perform concurrent tasks such as household chores, childcare, or exercise during online didactics [2, 3, 5]. Given the high rates of burnout in graduate medical training, these are not trivial considerations. Trainees also appreciate access to lecture recordings [2, 3, 5].

Although surveys of both university students and medical trainees have shown that many learners support a hybrid approach to educational activities with virtual attendance as a continued option, students also tend to view hybrid education more favorably than teachers [3–6]. In the post-pandemic university setting in which online class attendance remains an option, faculty have expressed disappointment in decreased live lecture attendance rates [7]. University faculty who have experienced teaching both in-person and online report feeling detached when lecturing remotely and a desire to return to fully in-person teaching in an ideal world [8]. However, in a post-pandemic graduate medical education survey of 88 clinical pediatric attendings, only 12% of faculty reported preferring entirely in-person education [5].

Studies of university students suggest that online class attendance is not necessarily inferior to in-person attendance. In a study of 273 students enrolled in two hybrid courses with the option to attend in-person or online, there was no statistically significant difference in student engagement scores, measured by class attendance and participation in active learning activities during lectures, or course performance between the two modes of attendance. However, there was a correlation between course performance and engagement (*r* = 0.38–0.65) in both groups of students [6]. Of the 189 survey respondents in this study who reported using the hybrid learning platform, 86% found at least one feature of the hybrid platform helpful to their learning, with lecture recordings being the most popular feature [6]. Another study of over 17,000 university students also showed lack of a clear relationship between course performance and mode of class attendance, whether attending in-person, attending online synchronously, or watching lecture recordings asynchronously [7]. Survey results from this study suggested that students were making conscious decisions about their mode of attendance based on their personal goals and needs; for example, some students reported choosing not to attend classes in-person because they felt more distracted in the lecture hall [7].

Multiple authors have published strategies for remote radiology education, and some radiology programs with residents working at multiple sites already relied on remote education prior to COVID-19 [1]. However, to our knowledge, the hybrid conference format in radiology residency and fellowship training programs has not been examined in the radiology education literature. The smaller setting and different learning needs involved in graduate medical education compared with undergraduate education require special considerations when designing hybrid conferences. An article in the *Harvard Business Review* described hybrid meetings as “vastly more complex” than completely in-person or virtual meetings, as it is all too easy for remote attendees to be treated as second-class participants when other attendees are physically together in a room [9]. In this article, we discuss the unique challenges associated with running an effective hybrid radiology conference and present strategies to support a positive experience for both learners and educators.

## The problem

The main challenge associated with hybrid meetings has been described as “primary room dominance,” referring to the diminished inclusion of remote participants relative to their in-person counterparts [10]. Remote participation—whether in purely virtual or in hybrid settings—presents distinct engagement challenges, such as the temptation of multitasking and difficulty maintaining focus when working in isolation. In hybrid settings, these challenges are compounded when remote participants are easily overlooked in favor of in-person participants [2–4, 11]. The challenges of maintaining engagement in hybrid settings build upon fundamental principles of meeting etiquette. Traditional meeting hygiene—avoiding multitasking, maintaining active participation, and keeping cameras on for remote participants—becomes even more critical in hybrid environments. For example, remote learners may find it difficult to signal that they want to answer a question posed by the educator, particularly if their camera is off [12]. The education literature recognizes a social dimension to student engagement, which involves communication and collaboration with both educator and peers [13].

The hybrid educational format not only affects students’ educational experience but also presents unique challenges to educators. Educators surveyed regarding hybrid teaching report challenges in dealing with the increased complexity of a hybrid setup and worry about unequal experiences among in-person versus remote learners [4]. Paying attention to both in-person and remote learners and navigating hybrid educational platforms, along with technological difficulties, increases educators’ mental load and fatigue [12]. In the post-pandemic education survey of 88 clinical pediatric attendings, only 29% of faculty reported feeling comfortable teaching virtually; 43% of faculty had no training in virtual education, and only 4% reported having extensive training in virtual education [5]. Faculty accustomed to giving traditional didactic in-person lectures must adapt their pedagogical methods, as remote learners tend to treat online lectures with the same passivity as if they were watching television [12]. In a hybrid education format where faculty lecture remotely, educators may also face difficulty in engaging the audience and gauging their response, which is similar to an online-only format [2, 3].

The education literature describes three critical dimensions of learning environment design: set, social, and epistemic design [8]. Successful hybrid education requires special attention to all three of these dimensions, which are interrelated. Set design encompasses the physical and technological infrastructure used for education, including tools, equipment, and space configuration [8]. Hybrid education requires specific technical capabilities that enable interaction between on-site and remote participants. Social design focuses on how human connections are built and maintained in the educational setting, for example by grouping students [8]. For hybrid education, social design presents unique challenges in facilitating interaction between on-site and remote participants, and creating a sense of belonging and presence across physical and virtual spaces. Epistemic design refers to the learning activities and pedagogical approaches that students participate in, such as collaborative problem-solving, assessment methods, and interactive components that facilitate knowledge construction and sharing [8]. Effective epistemic design in a hybrid conference format requires a foundation of optimal set and social design. In the following sections, we review specific strategies to optimize technological infrastructure, connection, and engagement in hybrid radiology education, addressing all three dimensions of learning environment design.

## Technology: setting the stage

### Basic requirements

Just as setup is key to success in a medical procedure, the first step to achieving an effective hybrid conference is setting up the conference space. Audio quality is perhaps the most critical technical element for successful hybrid conferences. The conference room should be equipped with high-quality microphones that can clearly capture both the lecturer and in-person participants’ questions and comments. Strategic microphone placement is essential—ceiling-mounted or table microphones should be positioned to cover all areas where in-person participants might speak from [14]. Having a hand-held or lapel microphone available as backup can help ensure remote participants can hear in-room discussion clearly [15]. Before each conference, audio levels should be tested from both in-person and remote perspectives, paying particular attention to [15, 16]:

1. Clarity of in-room participants’ voices for remote attendees.
2. Volume balance between lecturer and participant audio.
3. Elimination of echo or feedback.
4. Testing microphone coverage throughout the room.

Common audio pitfalls to watch for include:

1. Dead zones in the room where participant questions cannot be heard.
2. Echo from multiple devices being logged into the conference in the same room.
3. Feedback from speakers being too close to microphones.
4. Inconsistent volume levels between different speakers.

**Fig. 1:**
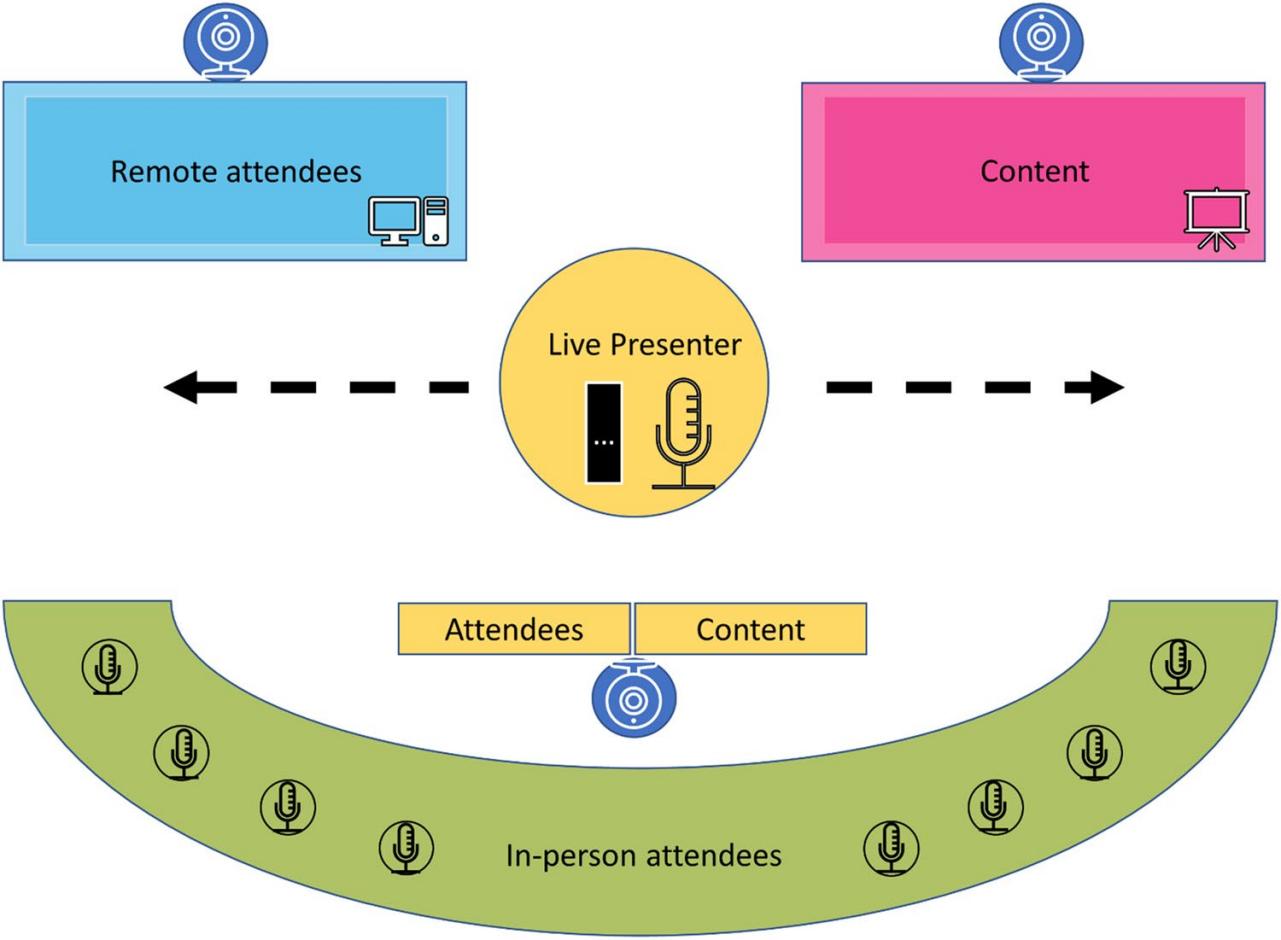
Diagram depicting a suggested room setup for a hybrid radiology educational conference. In-person attendees, who each have a microphone, face the presenter, a screen displaying the remote attendees’ video feed, and a separate screen displaying the educational material. The presenter has a monitor to view their presentation and another monitor to view remote attendees. Multiple cameras in the room allow remote attendees to view both the presenter and the in-person audience

Not only does the quality of the audiovisual equipment matter, but thoughtful positioning can also enhance the communal experience [9, 11]. In-room cameras should be set up to allow remote participants to see in-person participants while viewing the conference content [9, 11]. Not only can this increase a sense of inclusion among remote learners, but this also allows faculty who may be teaching remotely to view and engage with their audience. In addition, placing large monitors in the conference room to display remote participants’ faces and play their audio when they are speaking can also help increase their presence as full members of the conference [9]. A potential physical room setup for an effective hybrid radiology conference is diagrammed in Fig. 1.

Designating a conference moderator ahead of time can ease cognitive burdens on the educator and enhance inclusion of remote learners. A moderator can monitor remote participants raising their virtual hands or messaging in the chat function on the teleconferencing platform, relieving the educator of these responsibilities which require divided attention [11, 17, 18]. A moderator can help maintain equal engagement of in-person and remote learners [9]. For example, if the lecturer cannot view the remote participants, the moderator can identify participants to be called upon, or the moderator can assign remote participants to breakout rooms for group activities. For faculty who are lecturing remotely, an in-person moderator can help them engage with learners in the room. The moderator can also assist in handling technical issues that arise [17]. Anyone in the conference room with sufficient experience with the teleconferencing platform can serve as a moderator, including another faculty member, educational support staff, or even a trainee.

### Accessories to improve interactivity

Radiology faculty have many tools at their disposal for promoting interactivity during conferences. Audience response systems have the benefit of creating a low-stress interactive environment due to their anonymity and versatility of applications [1, 19]. They can be used to pose multiple-choice as well as open-ended questions, enable image interaction, and even allow learners to ask questions anonymously [1, 19]. Incorporating audience response systems requires more preparation from educators but provides the easiest method of including remote participants, as most of them are web-based. “Question and Answer (Q & A)” and survey applications are now incorporated in the most used teleconferencing platforms, eliminating the need for third-party software integration.

If the goal of a teaching session is to simulate the challenges of real-world case review, viewing static images in a presentation versus actual cross-sectional examinations is quite limited as subtle or atypical presentations of pathology can be difficult to capture in a few images [20]. Image stacks can be saved as videos that are played during a presentation to better mimic viewing a true cross-sectional examination. Alternatively, to even better simulate clinical practice, faculty can export full cases to web-based Digital Imaging and Communications in Medicine (DICOM) viewing platforms to provide trainees with scrollable interactive cases. Links to cases can be emailed to trainees beforehand or included in the lecture slides [20]. In-person and remote trainees can then be called on to review the cases or can be divided into groups for discussion. Instruction on use of educational technology should be incorporated into faculty development to empower faculty to make the most of available tools [5].

## Connection: establishing expectations

Establishing an “educational contract” by setting expectations and ensuring psychological safety is fundamental to adult learning [17]. It is especially important to delineate “rules of engagement” at the outset of a hybrid conference. By this point, most learners already know to mute themselves online when not speaking. However, the issue of turning video on or off is more controversial. While remote participants are often reluctant to turn on their video, this can be detrimental to the educational experience. A survey study of university students attending a hybrid course in-person, remotely with their camera on, or remotely with their camera off demonstrated significant differences in levels of multiple aspects of engagement between all three groups. In-person attendees reported the highest levels of engagement, while remote attendees with their camera on reported significantly higher levels of engagement compared with their camera-off counterparts [8]. One student commented that having the camera on makes remote students feel “obliged” to be attentive [8].

The invisibility of remote learners also impacts the experience of both the educator and other learners. The educator loses nonverbal cues from remote participants, hindering communication and the ability to gauge understanding and attention [11, 17]. Having remote participants represented as black boxes on a screen in contrast to in-person participants also impacts psychological safety. Psychological safety describes the extent to which meeting participants feel listened to and unafraid to speak up, and it requires all participants to give and receive equal attention [18]. From a practical standpoint, taking time to confirm the presence of remote participants without video distracts from the meeting, for example when a remote participant is called on but does not immediately respond. In the absence of video, it may be unclear if the participant’s lack of response is due to absence or technological issues [10]. Educators who are remote should also keep their video on [21].

Expectations for remote learners’ participation should be outlined, both to prevent the educator from neglecting the online audience and to prepare remote learners so that they are not caught off-guard when asked to speak. Setting a speaking order for both in-person and remote attendees can help ensure equal participation [10]. This has the added benefit of facilitating “warm-calling” learners, in which they know when they will be called upon soon [21]. In contrast to traditional “cold-calling,” this system provides an optimal balance of stress which is beneficial to student engagement and performance [21].

## Connection: preparation

The most effective educators will do some background research to know their audience. This includes the number of people expected to attend, their names, roles, levels of experience, and expectations. It is always wise to arrive 10–15 min ahead of schedule to meet attendees as they arrive on-site or on-screen. Familiarity with the audience allows the educator to incorporate this knowledge most effectively using the Knowles six principles of adult learning: (i) need to know why they have to learn this; (ii) need to incorporate their own experience; (iii) have to take responsibility for their learning; (iv) need context as to how this learning solves a particular problem; (v) seek problem-based learning; and (vi) are resistant to external prodding and must generate their own motivation [22]. Learners equally share the responsibility of understanding and applying these principles to their own educational journey. This preliminary work on both sides serves to maximize engagement.

## Engagement: interactivity and inclusivity

Hybrid education should go beyond simply streaming in-person lectures to remote learners [4, 23]. Active learning has been extensively studied as a tool for increasing student engagement and knowledge retention but is underutilized in graduate medical education. A proposed model for restructuring the traditional 1-h graduate medical education lecture involves interspersing shorter periods of didactic content delivery with active learning [23]. One radiology-specific study found that a 30/30 conference format, consisting of 30 min of didactics and 30 min of “hot seat” case conference, improved both short- and long-term knowledge retention as well as attention compared to traditional 60-min didactic lectures [24]. Some have argued that 10–15-min didactic lecturing separated by activity, such as case examples with participation, is even more effective for engagement and retention. In a hybrid conference, remote learners should be able to participate in interactive activities alongside their in-person colleagues [9]. It is important to actively solicit contributions from remote participants, as speaking up when remote is more difficult than in person [11].

Multiple strategies for interactive teaching have been presented in both the general medical education literature and the radiology literature, and these can be adapted to include both in-person and remote learners. The traditional case conference in which trainees take turns interpreting unknown cases involves the least preparation for faculty and is easily adapted to the hybrid education format, simply requiring faculty to call upon both remote and in-person trainees. Intentional use of digital tools to point out findings serves to strengthen training in optimal search patterns. As the American Board of Radiology returns to an oral examination format for the certifying examination, interactive case conferences for radiology trainees will be even more crucial.

To decrease the stress of answering questions in front of one’s peers, learners can be divided into smaller “buzz groups”—temporary small discussion groups of 2–4 people formed for quick, focused discussions on specific topics—that collaborate on questions throughout the lecture [23]. Peer instruction involves learners initially answering a question on their own and then collaborating with their peers to answer the same question; this strategy can be especially effective with learners of different levels, such as residents in different years [1, 23]. The most effective buzz group activities include focused questions; one example is to ask each buzz group a different question about the same case whereby all the salient points of the case end up being addressed. For these active learning methods involving grouping learners, careful consideration should be given to implementation methods. While virtual breakout rooms can facilitate remote participation, they may create technical challenges and participant anxiety, particularly when groups are randomly assigned. Pre-assigned groupings with clear objectives and expectations can help mitigate these concerns. Alternatively, remote participants can collaborate through shared digital whiteboards or be integrated with in-person participants who are logged into the conference on their personal devices, allowing for flexible interaction methods that suit different comfort levels. As discussed previously, a moderator can facilitate the creation of breakout rooms for the educator.

## Conclusion

Teaching conferences are an integral component of radiology training. It is important that radiology training programs recognize and address the challenges of hybrid conferences, as the hybrid format is likely here to stay. Success requires adequate investment in technological infrastructure, which is often overlooked but fundamental to effective implementation. The flexibility and accessibility of the hybrid model should not come at the expense of lower quality education. Both educators and learners must actively participate in this evolution—faculty through developing new teaching approaches and technical competencies, and learners through maintaining engagement and meeting established expectations for participation. The application of the strategies presented herein have promise to improve the educational value of hybrid conferences as well as educator satisfaction.

## Data Availability

No datasets were generated or analyzed during the current study.

## References

[1] Sivarajah RT, Curci NE, Johnson EM et al (2019) A review of innovative teaching methods. Acad Radiol 26:101–113 30929697 10.1016/j.acra.2018.03.025

[2] Tsyrulnik A, Gottlieb M, Coughlin RF et al (2021) Socially distanced, virtually connected: faculty and resident perceptions of virtual didactics. AEM Educ Train 5:e10617 34222751 10.1002/aet2.10617 PMC8239164

[3] Evans AZ, Adhaduk M, Jabri AR et al (2023) Is virtual learning here to stay? A multispecialty survey of residents, fellows, and faculty. Curr Probl Cardiol 48:101641 36773945 10.1016/j.cpcardiol.2023.101641 PMC9911980

[4] Mayer S, Abou Refaie R, Uebernickel F (2024) The challenges and opportunities of hybrid education with location asynchrony: implications for education policy. pfie. 10.1177/14782103231224507

[5] Roth LT, Mogilner L, Talib H et al (2023) Where do we go from here? Post-pandemic planning and the future of graduate medical education. Med Sci Educ 33:375–384 36778672 10.1007/s40670-023-01737-8 PMC9900559

[6] Adeel Z, Mladjenovic SM, Smith SJ et al (2023) Student engagement tracks with success in-person and online in a hybrid-flexible course. cjsotl-rcacea 14. 10.5206/cjsotlrcacea.2023.2.14482

[7] Kortemeyer G, Dittmann-Domenichini N, Schlienger C et al (2023) Attending lectures in person, hybrid or online-how do students choose, and what about the outcome? Int J Educ Technol High Educ 20:19 37008831 10.1186/s41239-023-00387-5 PMC10042582

[8] Raes A (2022) Exploring student and teacher experiences in hybrid learning environments: does presence matter? Postdigit Sci Educ 4:138–159 10.1007/s42438-021-00274-0 PMC8598934 40477740

[9] What it takes to run a great hybrid meeting. https://hbr-org.proxy.library.upenn.edu/2021/06/what-it-takes-to-run-a-great-hybrid-meeting. Accessed 8 Jan 2024

[10] Saatçi B, Akyüz K, Rintel S, Klokmose CN (2020) (Re)Configuring hybrid meetings: moving from user-centered design to meeting-centered design. Comput Support Coop Work 29:769–794 33230370 10.1007/s10606-020-09385-x PMC7675384

[11] Ellis R, Goodacre T, Mortensen N et al (2022) Application of human factors at hybrid meetings: facilitating productivity and inclusivity. Br J Oral Maxillofac Surg 60:740–745 35300882 10.1016/j.bjoms.2021.12.055 PMC8721917

[12] Raes A, Detienne L, Windey I, Depaepe F (2020) A systematic literature review on synchronous hybrid learning: gaps identified. Learn Environ Res 23:269–290

[13] Hietajärvi L (2022) Social engagement in distance-, remote-, and hybrid learning. J Online Learn Res 8:315–342

[14] Hamour O, Morrin H (2023) A quality improvement project to improve audio quality for remote attendees of a ward round at a London older adult psychiatric ward. BJPsych Open 9:S94–S94

[15] Marey A, Goubran S, Tarabieh K (2022) Refurbishing classrooms for hybrid learning: balancing between infrastructure and technology improvements. Buildings 12:738

[16] Basson S, Kanevsky D (2016) Improving audio conditions of audio and video conferences improving audio conditions of audio and video conferences. In: Technical Disclosure Commons. https://www.tdcommons.org/cgi/viewcontent.cgi?article=1415&context=dpubs_series. Accessed 22 Jan 2025

[17] Ohnigian S, Richards JB, Monette DL, Roberts DH (2021) Optimizing remote learning: leveraging zoom to develop and implement successful education sessions. JMECD 8:23821205211020760 10.1177/23821205211020760 PMC8243086 34263055

[18] Constantinides M, Quercia D (2022) The future of hybrid meetings. In: Kun AL, Shaer O, Boll S, Fox S, Raval N, Wilson ML (eds) 2022 Symposium on human-computer interaction for work. ACM, New York, NY, USA, pp 1–6

[19] Richardson ML, Shaffer K, Amini B, Spittler NLJ (2020) Advanced, interactive, image-based education: technology and pedagogy. Curr Probl Diagn Radiol 49:74–81 31300178 10.1067/j.cpradiol.2019.06.003

[20] Sugi MD, Kennedy TA, Shah V, Hartung MP (2021) Bridging the gap: interactive, case-based learning in radiology education. Abdom Radiol (NY) 46:5503–5508 34086093 10.1007/s00261-021-03147-z PMC8175917

[21] Said JT, Schwartz AW (2021) Remote medical education: adapting Kern’s curriculum design to tele-teaching. Med Sci Educ 31:805–812 33520396 10.1007/s40670-020-01186-7 PMC7833892

[22] Knowles MS, Holton III EF, Swanson RA, Robinson PA (2020) The adult learner: the definitive classic in adult education and human resource development, 9th ed. Routledge, London

[23] Cooper AZ, Richards JB (2017) Lectures for adult learners: breaking old habits in graduate medical education. Am J Med 130:376–381 27908794 10.1016/j.amjmed.2016.11.009

[24] Pamarthi V, Grimm L, Johnson K, Maxfield C (2019) Hybrid interactive and didactic teaching format improves resident retention and attention compared to traditional lectures. Acad Radiol 26:1269–1273 31085099 10.1016/j.acra.2019.02.018

